# The impact of chronic mild hypoxia on cerebrovascular remodelling; uncoupling of angiogenesis and vascular breakdown

**DOI:** 10.1186/s12987-021-00284-x

**Published:** 2021-11-17

**Authors:** Sebok K. Halder, Richard Milner

**Affiliations:** San Diego Biomedical Research Institute, 10865 Road to the Cure, Suite 100, San Diego, CA 92121 USA

**Keywords:** Brain, Chronic mild hypoxia, Blood–brain barrier integrity, Fibrinogen, Angiogenesis, Vascular leak

## Abstract

**Background:**

Chronic mild hypoxia (CMH, 8% O_2_) stimulates robust vascular remodelling in the brain, but it also triggers transient vascular disruption. This raises the fundamental question: is the vascular leak an unwanted side-effect of angiogenic remodelling or is it a pathological response, unrelated to endothelial proliferation, in which declining oxygen levels trigger endothelial dysfunction?

**Methods:**

To answer this question, mice were exposed to CMH (8% O_2_) for periods up to 14 days, after which, brain tissue was examined by immunofluorescence (IF) to determine which type of blood vessel (arteriole, capillary or venule) was most commonly associated with endothelial proliferation and vascular leak and how this correlated with tight junction protein expression. Vascular perfusion was examined using DiI. Data were analysed using one-way analysis of variance (ANOVA) followed by Tukey’s multiple comparison post-hoc test.

**Results:**

The following was observed: (1) most endothelial proliferation and extravascular fibrinogen leak occurred in capillaries and to a lesser degree in venules, (2) much to our surprise, endothelial proliferation and extravascular fibrinogen leak never colocalized, (3) interestingly however, endothelial proliferation was strongly associated with an intravascular fibrinogen staining pattern not seen in stable blood vessels, (4) DiI perfusion studies revealed that angiogenic vessels were adequately perfused, suggesting that fibrinogen retention in angiogenic vessels is not due to temporary closure of the vessel, but more likely because fibrinogen is retained within the vessel wall, (5) bromodeoxyuridine (BrdU) labelling as a means to more permanently label proliferating endothelial cells, confirmed lack of any connection between endothelial proliferation and extravascular fibrinogen leak, while (6) in contrast, proliferating microglia were detected within extravascular leaks.

**Conclusions:**

Taken together, our findings support the concept that in the short-term, hypoxia-induced endothelial proliferation triggers transient fibrinogen deposition within the walls of angiogenic blood vessels, but no overt vascular leak occurs in these vessels. Importantly, endothelial proliferation and extravascular fibrinogen leaks never co-localize, demonstrating that extravascular leak is not an unwanted side-effect of angiogenic endothelial proliferation, but rather a dysfunctional vascular response to hypoxia that occurs in a distinct group of non-angiogenic blood vessels.

## Introduction

Cerebral blood vessels are unique in having high electrical resistance and low permeability, which protects sensitive cells in the brain neuropil from potentially disruptive and harmful blood components [1–3]. This low permeability property of cerebral blood vessels is commonly referred to as the blood–brain barrier (BBB), whose molecular basis depends on a combination of inter-endothelial tight junction proteins, vascular basal lamina extracellular matrix (ECM) proteins, and the influence of astrocyte end-feet and pericytes [4–7].

While the brain constitutes only 2% of the dry weight of the body, it receives more than 20% of cardiac output and utilizes more than 25% of total glucose uptake, demonstrating that the brain is a highly metabolic organ, requiring a disproportionately high blood supply [8]. Because of these high demands, cerebral blood vessels are acutely responsive to changes in environmental conditions in order to meet the metabolic requirements of neural tissue. When exposed to low oxygen (hypoxic) conditions, the body launches several adaptive mechanisms which strive to maintain a constant supply of oxygen and nutrients to the brain. In the short term, acute adaptive responses include increased heart rate, blood pressure and ventilation rate, as well as an increased cerebral blood flow that is mediated by vasodilation of cerebral blood vessels, also known as cerebral autoregulation [8, 9]. If the hypoxia state is maintained, then over days to weeks, a series of chronic adaptations occur, which include enhanced hematocrit, to increase the oxygen carrying capacity of the blood, as well as active remodelling of the cerebral vasculature, which increases the vessel density and complexity, so that every cell in the brain is physically closer to a blood vessel [8, 9]. In the laboratory, this point is well illustrated in the chronic mild hypoxia (CMH) model, which demonstrates that when rodents are exposed to mild hypoxia (typically 8–10% O_2_) for prolonged periods of time, their cerebral blood vessels mount a strong vascular remodelling response, resulting in greater than 50% enhancement of vessel density over a period of two weeks [8, 9].

Using this model, we recently showed that in addition to promoting a marked angiogenic remodelling response, CMH also promotes transient vascular leak in cerebral blood vessels [10]. Interestingly, we found that the extent of vascular leak varies markedly between different brain regions, and that within different regions, the degree of vascular leak correlates closely with the degree of angiogenic remodelling. This raises the fundamental question: why does CMH trigger vascular leak? Is it an unwanted side-effect of physiological angiogenic remodelling or is it part of a pathological response, unrelated to endothelial proliferation, in which declining oxygen levels trigger endothelial dysfunction? To address this fundamental question, we performed a detailed spatial and temporal analysis of the vascular remodelling and leak that occurs in the brains of CMH-exposed mice, to (i) define which type of cerebral vessel (arterioles, capillaries or venules) is most implicated in CMH-induced endothelial proliferation and vascular leak, and (ii) determine how closely these two events are related.

## Materials and methods

### Animals

The studies described were reviewed and approved by the Explora Biolabs Institutional Animal Care and Use Committee at San Diego Biomedical Research Institute (SDBRI). Wild-type female and male C57BL6/J mice obtained from Jackson Laboratories were maintained under pathogen-free conditions in the closed breeding colony of SDBRI.

### Chronic hypoxia model

Female and male wild-type C57BL6/J mice, 8–10 weeks of age, were housed 4 to a cage, and placed into a hypoxic chamber (Biospherix, Redfield, NY) maintained at 8% O_2_ for periods up to 14 days. Littermate control mice were kept in the same room under similar conditions except that they were kept at ambient sea-level oxygen levels (normoxia, approximately 21% O_2_ at sea-level) for the duration of the experiment. Every few days, the chamber was briefly opened for cage cleaning and food and water replacement as needed.

### Immunohistochemistry and antibodies

Immunohistochemistry was performed on 10 μm frozen sections of cold phosphate buffer saline (PBS) perfused tissues as described previously [11]. Antibodies used in this study included: rat monoclonal antibodies reactive for CD31 (clone MEC13.3; 1:300) and Mac-1 (clone M1/70; 1:50) from BD Pharmingen (La Jolla, CA), laminin α1 (clone 200; 1:10,000), a kind gift from Dr. Lydia Sorokin of University of Munster, Germany, hamster monoclonal antibodies against CD31 (clone 2H8; 1:500) from Abcam (Cambridge, MA) and β3 integrin (clone 2C9.G2; 1:100) from BD Pharmingen, mouse monoclonal antibodies against anti-Ki67 (1:500) from Vector Laboratories (Burlingame, CA) and α-SMA-Cy3 conjugate (clone 1A4 (1: 2000) from Sigma, St. Louis, MO), rabbit polyclonal antibodies reactive to occludin, ZO-1, and claudin-5 (all  1:2000 from Invitrogen, Carlsbad, CA), fibrinogen (1:2000 from Millipore, Temecula, CA) and the anti-laminin α1 subunit (1:10,000, a kind gift from Dr. Takako Sasaki of Oita University, Japan). Secondary antibodies used (all at 1:500) included Cy3-conjugated anti-rabbit, anti-rat and anti-mouse and Cy5-conjugated anti-rabbit and anti-mouse from Jackson Immunoresearch, (West Grove, PA) and Alexa Fluor 488-conjugated anti-rat, anti-mouse, anti-hamster, and anti-rabbit from Invitrogen (Carlsbad, CA). In some studies, instead of using fibrinogen to indicate extravascular leak, we used Cy3-conjugated anti-mouse to detect leaked mouse IgG.

### DiI labelling of vasculature

Blood vessel patency was examined by cardiac perfusion with the lipophilic dye 1,1′-dioctadecyl-3,3,3′,3′-tetramethylindocarbocyanine perchlorate (DiI) as previously described [12]. Briefly, mice were cardiac perfused with an ice-cold solution of 12 µg/ml DiI for 2 min, followed by perfusion with cold PBS for 2 min. Brains were harvested, mounted in OCT compound and stored at − 80 °C. 10 μm frozen sections were then prepared and visualized immediately under fluorescent microscopy and images captured by digital camera. The same sections were then further processed for staining with CD31/fibrinogen or CD31/Ki67 to examine the relationship between blood vessels and perfusion as well as fibrinogen deposition and endothelial proliferation.

### BrdU incorporation studies

Cell proliferation in the brain was examined using the bromodeoxyuridine (BrdU) labelling approach, as detailed in the manufacturer’s instructions (Roche molecular biochemicals, Indianapolis, IN). Briefly, mice exposed to 4 days chronic mild hypoxia (CMH) received daily intraperitoneal (i. p.) injections of BrdU (200 µl of stock solution) for the duration of the experiment. After brain harvest and embedding in OCT compound, 10 μm frozen sections were then incubated with mouse anti-BrdU antibodies, followed by anti-mouse-Cy3 as described in the manufacturer’s instructions.

### Image analysis

Images were taken using a 5×, 10× or 20× objective on a Zeiss Imager M1.m fluorescent microscope (Thornwood, NY). For each antigen in all analyses, images of at least three randomly selected areas were taken at 10× or 20× magnification per tissue section and three sections per brain analyzed to calculate the mean for each animal (n = 4 mice per group). For each antigen in each experiment, exposure time was set to convey the maximum amount of information without saturating the image and was maintained constant for each antigen across the different experimental groups. The number of blood vessels showing the intravascular or extravascular (leaking) fibrinogen pattern per field of view (FOV) was quantified by capturing images and performing manual counts. Endothelial proliferation was quantified by counting the number of CD31/Ki67 dual-positive cells per FOV. The number of blood vessels showing loss of the tight junction proteins ZO-1, occludin and claudin-5 was quantified by capturing images and performing manual counts. Each experiment was performed with 4 different animals per condition, and the results expressed as the mean ± SEM. Statistical significance was assessed using one-way analysis of variance (ANOVA) followed by Tukey’s multiple comparison post-hoc test, in which p < 0.05 was defined as statistically significant.

## Results

### Chronic mild hypoxia (CMH) induces transient vascular leak predominantly in capillaries

**Fig. 1:**
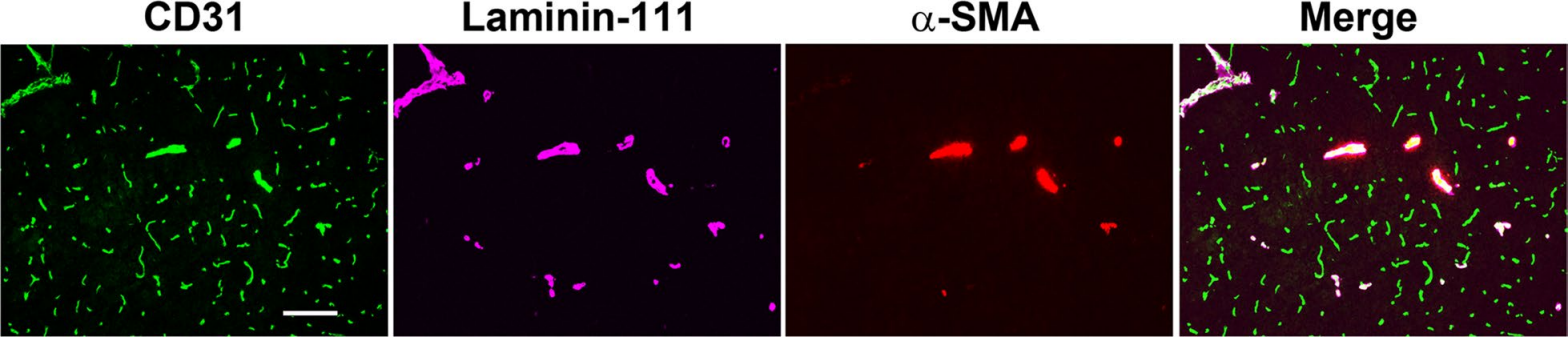
Distinguishing the three major types of blood vessel. Frozen mouse brain sections were triple-labelled with the endothelial cell marker CD31 (AlexaFluor-488), laminin-111 (Cy5), and α-SMA (Cy3). Scale bar = 100 μm. Note that CD31 labels all vessel types (capillaries, arterioles and venules), laminin-111 labels only the larger diameter vessels (including arterioles and venules), but not capillaries, and the smooth muscle cell marker α-SMA labels specifically arterioles

**Fig. 2:**
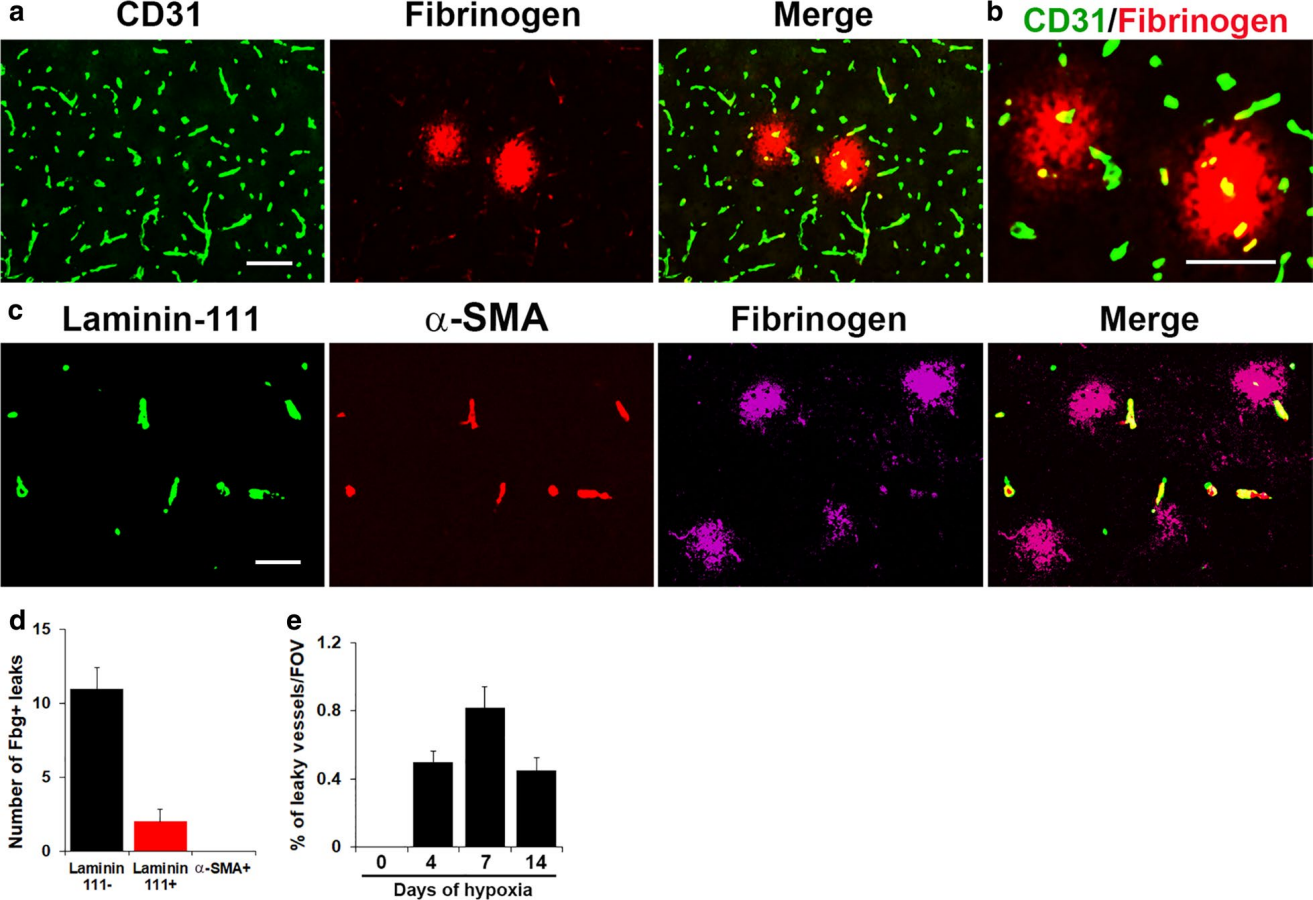
Leaky vessels are predominantly capillaries. Frozen brain sections taken from mice exposed to 4 days hypoxia (8% O_2_) were dual-labelled for CD31 (AlexaFluor-488) and fibrinogen (Cy3) (**A**, **B**), or triple-labelled for laminin-111 (AlexaFluor-488), α-SMA (Cy3), and fibrinogen (Cy5) (**C**). Scale bars = 100 μm. **D** Quantification of the number of capillaries (laminin-111-negative), venules (laminin-111^+^/α-SMA-negative) or arterioles (α-SMA^+^)/FOV that showed extravascular fibrinogen leak after 4 days hypoxia. **E** Time-course of the % of vessels showing vascular leak. All results are expressed as the mean ± SEM (n = 4 mice/group). Note that CMH-induced vascular leak occurs predominantly in capillaries (laminin-111 negative), rarely in venules, but never in arterioles

In this study we used a panel of markers to identify the three main types of cerebral blood vessel; the pan-endothelial marker CD31 which labels all vessel types (capillaries, arterioles and venules), laminin-111, which has been shown to identify arterioles and venules but not capillaries [13], and the smooth muscle cell marker α-SMA which specifically labels arterioles (Fig. 1) [14]. In all studies, mice were fully perfused to remove all blood (including fibrinogen) before tissues were collected. To determine which type of cerebral blood vessel is disrupted by chronic mild hypoxia (CMH, 8% O_2_), we performed CD31/fibrinogen dual-immunofluorescence (dual-IF) on frozen brain sections taken from mice exposed to 4 days CMH (Fig. 2A and B). We defined vascular leak as fibrinogen staining that extends beyond the confines of the CD31^+^ blood vessel wall, as illustrated in Fig. 2A and at high power magnification in Fig. 2B. CD31/fibrinogen dual-IF revealed that vascular leak was most often associated with the smallest caliber vessels, namely capillaries, but much less commonly with the larger caliber venules and arterioles. Laminin-111/α-SMA/fibrinogen triple-IF confirmed that the majority of vascular leak occurred in vessels that stained negative for laminin-111, namely capillaries, while a small fraction of leaks were associated with laminin-111^+^ vessels but never with α-SMA^+^ arterioles (Fig. 2C). As quantified in Fig. 2D, this demonstrates that CMH-induced vascular leak occurs predominantly in capillaries (laminin-111 negative), rarely in venules, but never in arterioles. As shown in Fig. 2E, quantification revealed that the % of vessels showing vascular leak peaked after 7 days CMH (0.82 ± 0.12%) and declined at later timepoints. In this study, while we focused our analysis primarily in the brainstem region, these findings were consistent in all regions of the brain examined.

### CMH-induced endothelial proliferation occurs predominantly in capillaries and venules

**Fig. 3:**
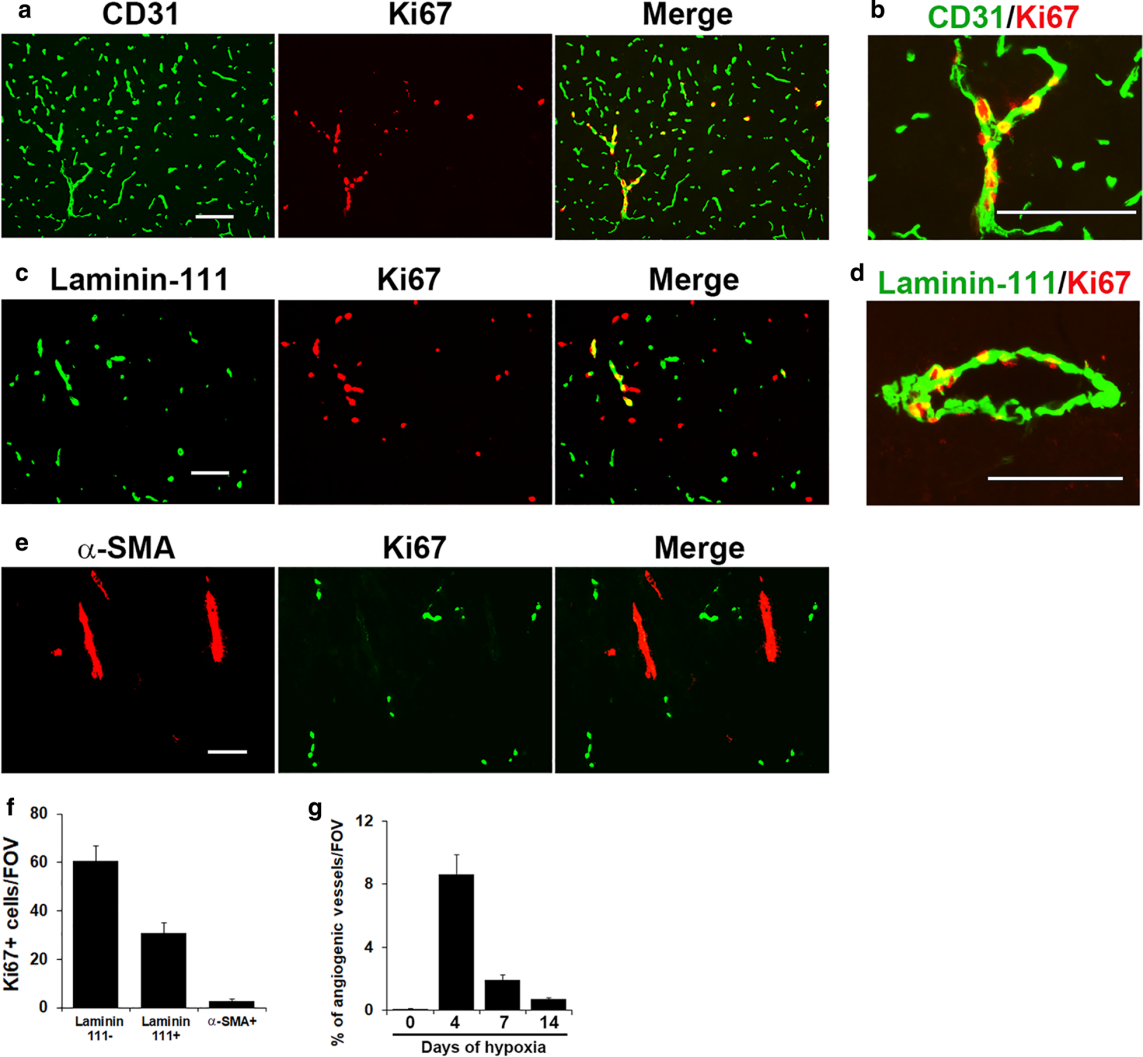
CMH-induced endothelial proliferation occurs predominantly in capillaries and venules. Frozen brain sections taken from mice exposed to 4 days hypoxia (8% O_2_) were dual-labelled for CD31 (AlexaFluor-488) and Ki67 (Cy3) (**A**, **B**), laminin-111 (AlexaFluor-488) and Ki67 (Cy3) (**C**, **D**), or α-SMA (Cy3) and Ki67 (AlexaFluor-488) (**E**). Scale bars = 100 μm. **F** Quantification of the number of proliferating endothelial cells (CD31^+^/Ki67^+^ cells)/FOV after 4 days hypoxia. **G** Time-course of the % of total vessels that are angiogenic. Results are expressed as the mean ± SEM (n = 4 mice/group). Note that the majority (approximately two-thirds) of proliferating endothelial cells were located in capillaries (laminin-111 negative vessels), and one-third in venules, with very few in arterioles

To determine in which type of blood vessel CMH stimulates endothelial proliferation, we next examined brain sections taken from mice exposed to 4 days CMH, the time-point of maximal endothelial proliferation. Dual-IF of CD31 with the proliferation marker Ki67 showed that many Ki67^+^ cells were found in the smallest diameter vessels (capillaries) but some were also present in larger diameter vessels (Fig. 3A and B). Interestingly, in large diameter vessels, some Ki67^+^ cells were also found in laminin-111^+^ vessels (Fig. 3C and D), but only very rarely in α-SMA^+^ arterioles (Fig. 3E). This demonstrates that CMH stimulates endothelial proliferation predominantly in capillaries and venules. Quantification of laminin-111/Ki67 dual-IF revealed that the majority (approximately two-thirds) of proliferating endothelial cells were located in capillaries (laminin-111 negative vessels), and one-third in venules (Fig. 3F). As shown in Fig. 3G, quantification revealed that the % of total vessels that were angiogenic peaked after 4 days CMH (8.6 ± 1.2%) and declined at later timepoints. Of note, groups of proliferating cells were often observed in tight clusters, lined up in angiogenic capillaries or venules (Fig. 3B and D). As the studies presented were performed in the brains of female mice, we also examined the extent of endothelial proliferation and vascular leak in male mice and observed very similar responses (not shown).

### Endothelial proliferation is associated with an intravascular pattern of fibrinogen staining but not with extravascular leak

**Fig. 4:**
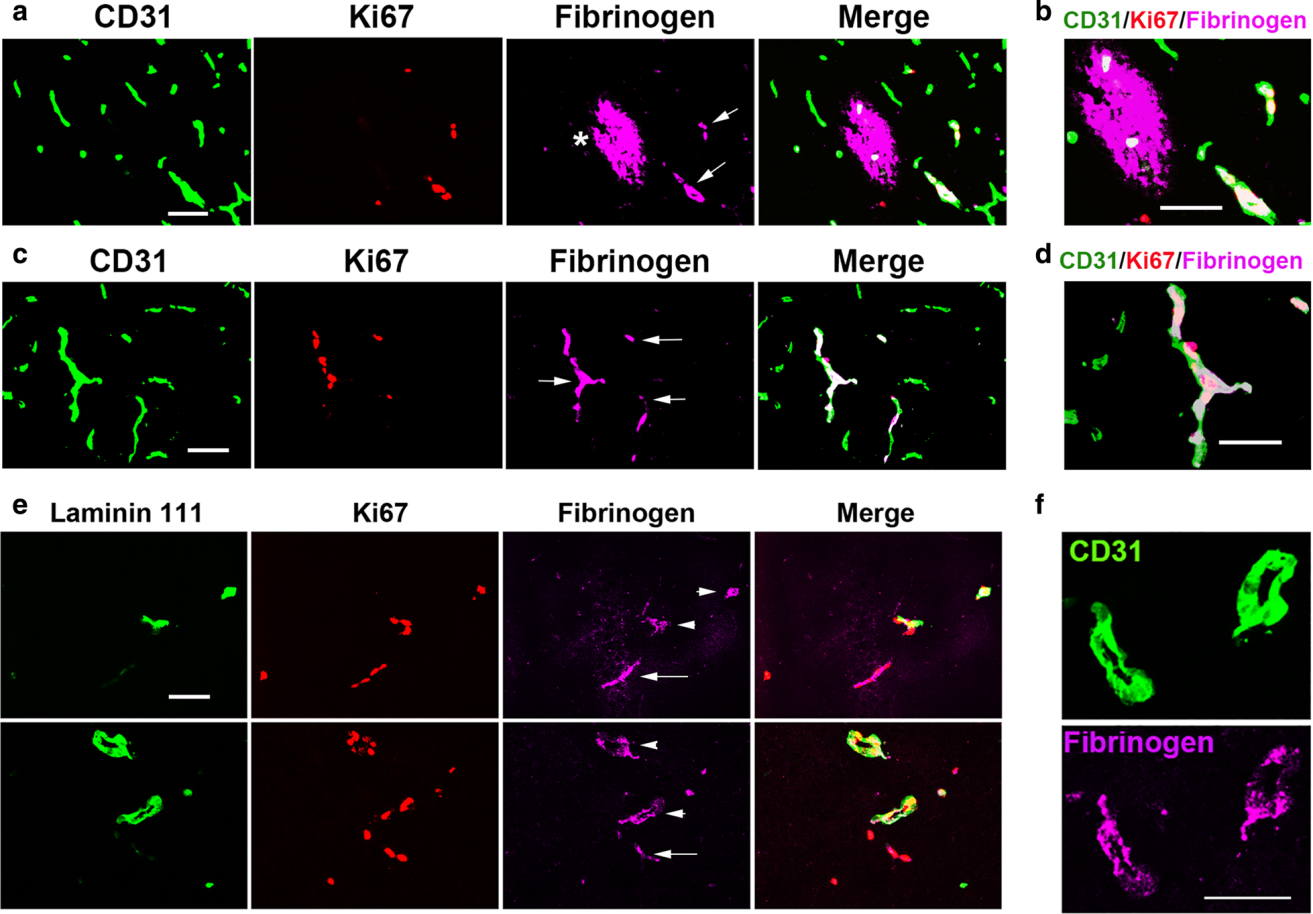
Endothelial proliferation is associated with an intravascular pattern of fibrinogen staining but not with vascular leak. Frozen brain sections taken from mice exposed to 4 days hypoxia (8% O_2_) were triple-labelled for CD31 (AlexaFluor-488), Ki67 (Cy3) and fibrinogen (Cy5) (**A–D**), or laminin-111 (AlexaFluor-488), Ki67 (Cy3) and fibrinogen (Cy5) (**E–F**). Scale bars = 50 μm. Note that proliferating (Ki67^+^) endothelial cells were never associated with extravascular leak (asterisk), but they were strongly associated with an intravascular fibrinogen staining pattern (arrows). Vessels that were neither angiogenic nor leaky showed no fibrinogen staining of any kind. In panel **E**, also note that intravascular fibrinogen staining was found both in angiogenic laminin-111-negative capillaries (arrows) and laminin-111^+^ venules (arrowheads) and appeared to line the walls of blood vessels (**F**)

As our findings demonstrate that CMH induces most endothelial cell proliferation and vascular leak in the same type of vessel (capillaries), this raises the question: is it possible that when proliferating endothelial cells uncouple during angiogenic remodelling, this triggers transient vascular leak? To answer this question, we closely examined the spatial and temporal relationship between endothelial proliferation, a key early step in the angiogenic response, and vascular leak, by performing CD31/Ki67/fibrinogen triple IF on brain sections taken from mice exposed to 4 days CMH. We expected to see a close spatial connection between endothelial proliferation and extravascular fibrinogen leak. However, much to our surprise, as shown in Fig. 4A and at higher power in Fig. 4B, in all brain regions examined, Ki67+ endothelial cells were never associated with the extensive “flash” of extravascular leak (asterisk), but they were strongly associated with an intravascular fibrinogen staining pattern (Fig. 4A–D), in which despite all blood being flushed out during terminal perfusion, fibrinogen remained confined within the blood vessel wall (arrows). This was in stark contrast to vessels that were neither angiogenic nor leaky, which showed no fibrinogen staining of any kind. Quantification analysis revealed that endothelial proliferation was always associated with intravascular fibrinogen deposition, but never associated with extravascular fibrinogen leak. These findings raise the related question: is the intravascular fibrinogen staining pattern specific to capillaries or does it also occur in venules? As illustrated in Fig. 4E, laminin/Ki67/fibrinogen triple-IF showed that intravascular fibrinogen staining was found both in angiogenic laminin-111-negative capillaries (arrows) and laminin-111^+^ venules (arrowheads). Analysis after 4 days CMH revealed that the intravascular fibrinogen pattern was far more common than the extravascular leak pattern. Most notably, the presence of Ki67^+^ cells within blood vessels strongly co-localized with the intravascular staining pattern, but never with the extravascular leak pattern. These findings demonstrate that during the process of vascular remodelling, angiogenic capillaries retain the intravascular fibrinogen that is normally flushed out during terminal perfusion. The question is why? We considered three main possible explanations. First, as a protective response to prevent catastrophic vascular leak during angiogenesis, it is possible that blood flow through the remodelling vessel is temporarily closed off, by contraction of the arteriolar pre-capillary sphincter. Second, as proliferating endothelial cells become more activated during angiogenic remodelling, this stimulates transient fibrinogen binding to the vessel wall, possibly via induced expression of the endothelial fibrinogen receptor αvβ3 integrin, known to be upregulated on angiogenic cerebral vessels [15, 16]. Third, during angiogenic remodelling, endothelial cells transiently separate from neighbouring cells as they proliferate and migrate to extend the vascular network; thus the intravascular fibrinogen staining pattern may represent an early stage of vascular leak in which fibrinogen has passed partially through the vessel wall, potentially on its way to becoming a full-blown extravascular leak.

**Fig. 5:**
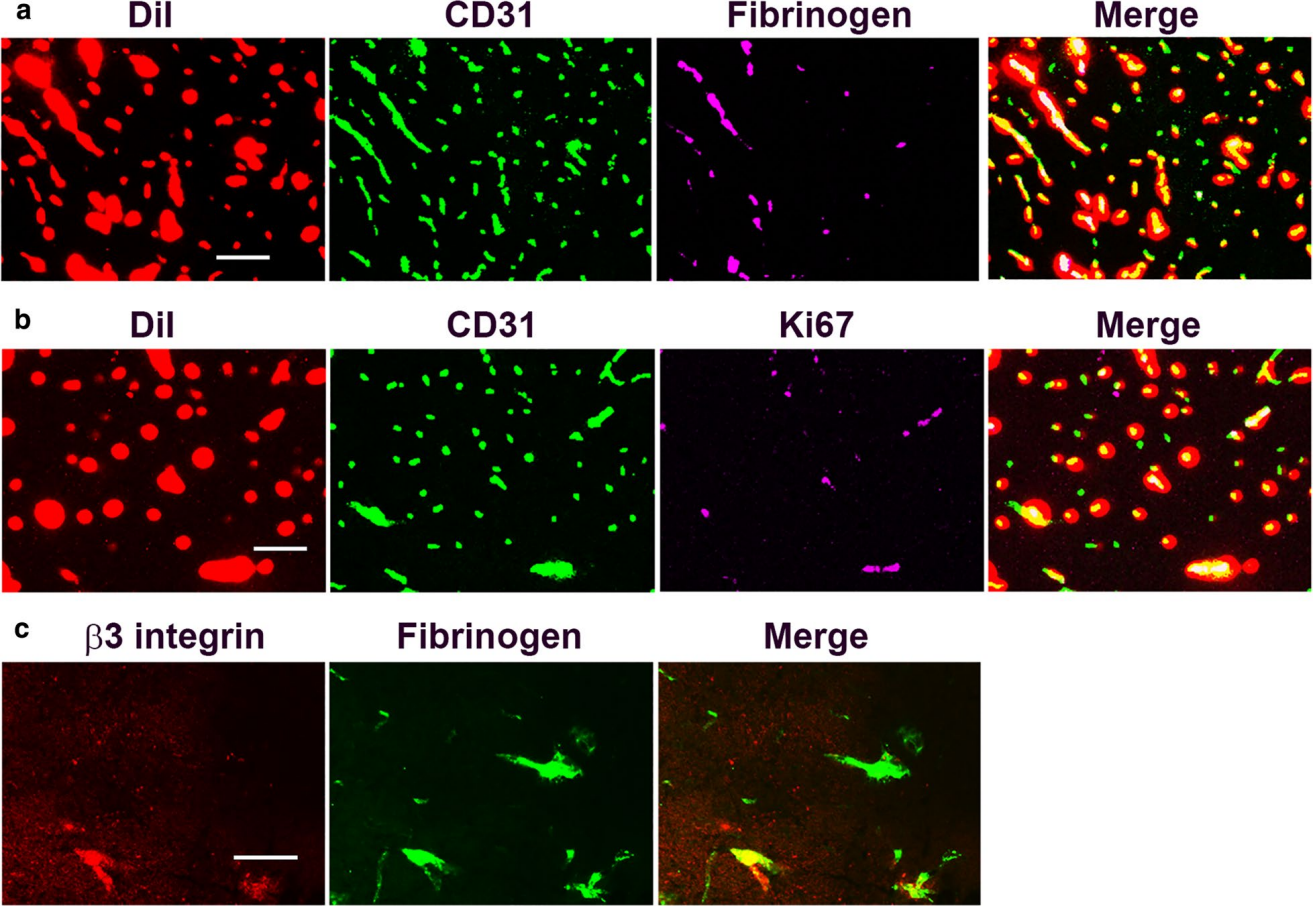
Analysis of intravascular fibrinogen staining. **A**,**B** Mice exposed to 4 days hypoxia (8% O_2_) were perfused with DiI (red channel) and frozen brain sections subsequently dual-labelled for CD31 (AlexaFluor-488) and fibrinogen (Cy5) (**A**), or CD31 (AlexaFluor-488) and Ki67 (Cy5) (**B**). **C** Frozen brain sections taken from mice exposed to 4 days hypoxia (8% O_2_) were dual-labelled for β3 integrin (Cy3) and fibrinogen (AlexaFluor-488). Scale bar = 100 μm (**A**, **B**) and 50 μm (**C**). Note that blood vessels showing the intravascular fibrinogen pattern or containing Ki67^+^ endothelial cells, stained strongly positive for DiI. Also note that while β3 integrin and fibrinogen occasionally co-localized, often they did not

To address the first possibility, namely that blood flow is temporarily closed off when vessels are actively remodelled, we perfused mice that had been exposed to 4 days CMH with the lipophilic dye DiI, which permits visualization of the entire perfused vascular network. If blood vessels are temporarily closed off during vascular remodeling, we predicted that blood vessels showing intravascular fibrinogen staining or the presence of Ki67^+^ endothelial cells would show no DiI signal. However, as shown in the DiI/CD31/fibrinogen and DiI/CD31/Ki67 triple-IF images shown in Fig. 5A and B respectively, blood vessels that displayed the intravascular fibrinogen pattern or contained Ki67^+^ endothelial cells, also stained strongly positive for DiI, indicating that actively remodeling cerebral blood vessels are not closed off but continue to transmit blood during the angiogenic process. To address the second possibility, we reasoned that if upregulated expression of αvβ3 integrin was responsible for the intravascular fibrinogen pattern, then vascular β3 integrin and intravascular fibrinogen would show strong co-localization. However, as shown in Fig. 5C, β3 integrin/fibrinogen dual-IF revealed that while the two markers occasionally co-localized, often they did not, suggesting that endothelial induction of αvβ3 integrin is not required for the intravascular fibrinogen pattern to be manifest.

**Fig. 6:**
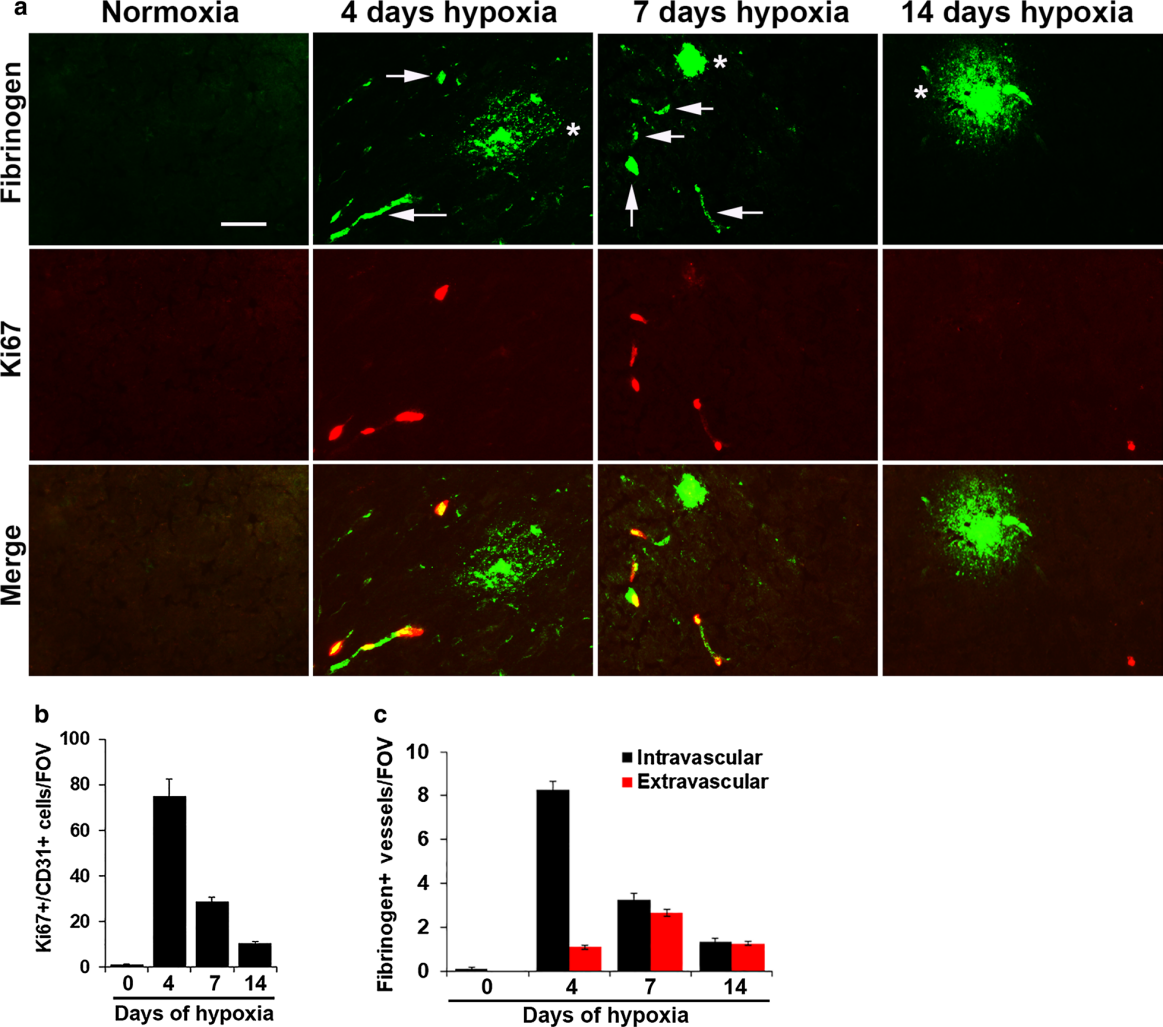
Examining the time course of CMH-induced endothelial proliferation and fibrinogen staining patterns. **A** Frozen brain sections taken from mice exposed to hypoxia (8% O_2_) for 4, 7 or 14 days were stained for fibrinogen (AlexaFluor-488) and the proliferation marker Ki67 (Cy3). Scale bar = 50 μm.  Note the intravascular fibrinogen staining pattern (arrows) and the extravascular fibrinogen leaks (asterisks). **B**, **C** Quantification of the number of proliferating endothelial cells (CD31^+^/Ki67^+^ cells)/FOV (**B**) or vessels showing intravascular/extravascular fibrinogen staining (**C**) at different timepoints. All results are expressed as the mean ± SEM (n = 4 mice/group). Note the close correlation between the rate of endothelial proliferation and number of vessels showing intravascular fibrinogen staining, which both peaked at day 4 and declined thereafter. The number of vessels showing extravascular fibrinogen leak peaked at day 7 but was comparatively small at all timepoints

At the outset of these studies, we considered that the intravascular fibrinogen pattern associated with angiogenic capillaries most likely represents an early stage of extravascular leak. If this is true, one would predict that over time, most of the vessels showing the intravascular pattern would evolve into the extravascular pattern, resulting in decreased numbers of vessels showing the intravascular fibrinogen pattern and increased number showing extravascular leak at later timepoints. To test this idea, we extended our observations made at the 4-day timepoint, by examining these events at the later timepoints of 7 and 14 days CMH. As expected, normoxic brain tissue contained no proliferating cells or any fibrinogen staining (Fig. 6A), but after 4 and 7 days CMH, both intravascular (arrows) and extravascular (asterisks) fibrinogen staining patterns could be seen, with the intravascular pattern strongly associated with Ki67^+^ proliferating endothelial cells. By 14 days CMH, both types of fibrinogen staining pattern and Ki67^+^ cells had dropped to much lower levels. Quantification revealed that brains from 4 days CMH mice showed the highest number of proliferating endothelial (Ki67^+^/CD31^+^) cells (Fig. 6B) and vessels showing intravascular fibrinogen staining, but very few vessels with extravascular fibrinogen leak (Fig. 6C). After 7 days CMH, the number of proliferating endothelial cells and vessels with intravascular fibrinogen staining had declined in parallel, while the number of vessels showing extravascular fibrinogen leak had slightly increased but was still relatively low compared to the number showing intravascular fibrinogen staining at day 4. By 14 days CMH, the number of proliferating endothelial cells and vessels showing either intravascular or extravascular fibrinogen leak staining had all declined to much lower levels. These data demonstrate that while endothelial proliferation is strongly associated with the intravascular fibrinogen staining pattern, both spatially and numerically, they argue against the concept that blood vessels showing this pattern transform into vessels displaying extravascular fibrinogen leak.

### Confirming the lack of connection between endothelial proliferation and vascular leak

**Fig. 7:**
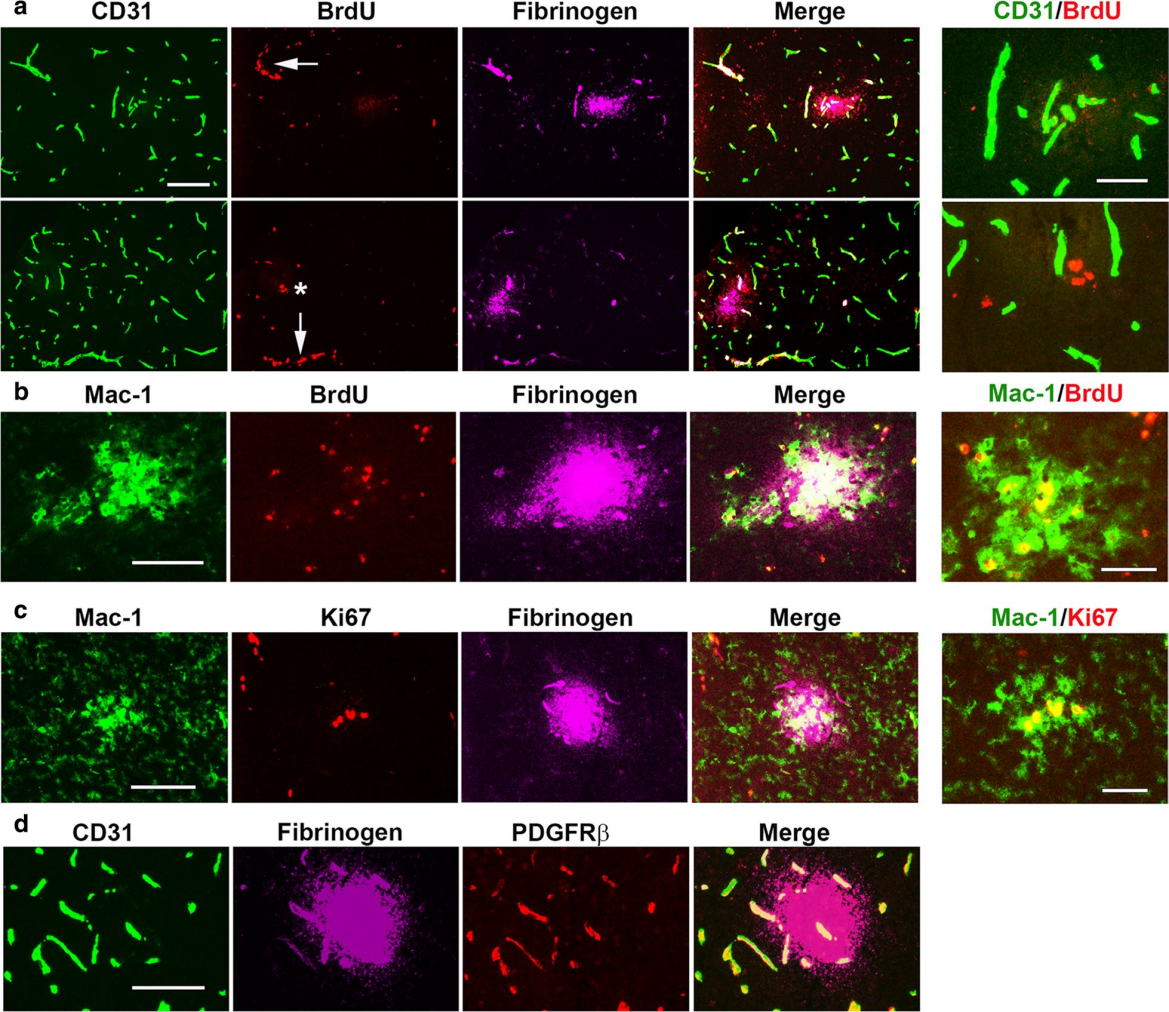
Endothelial and microglial proliferation are associated with intravascular and extravascular patterns of fibrinogen staining, respectively. Frozen brain sections taken from mice exposed to 4 days hypoxia (8% O_2_) were triple-labelled for CD31 (AlexaFluor-488), BrdU (Cy3) and fibrinogen (Cy5) (**A**), Mac-1 (AlexaFluor-488), BrdU (Cy3) and fibrinogen (Cy5) (**B**), Mac-1 (AlexaFluor-488), Ki67 (Cy3) and fibrinogen (Cy5) (**C**), or CD31 (AlexaFluor-488), fibrinogen (Cy5) and PDGFRβ (Cy3) (**D**). Scale bar = 50 μm. High power images on extreme right, scale bar = 25 μm. Note that proliferating (BrdU^+^) endothelial cells were never associated with extravascular leak, but they were strongly associated with the intravascular fibrinogen staining pattern (arrows). In contrast, proliferating microglia were strongly associated with extravascular fibrinogen leak

Because we observed that proliferating endothelial cells were never associated with extravascular fibrinogen leak, there remained the question of whether endothelial proliferation eventually leads to subsequent vascular leak or whether the leak occurs in an entirely distinct group of vessels that are non-angiogenic. We reasoned that the most likely explanation that these two markers never co-localize is that their expression occurs sequentially. In other words, endothelial proliferation, as defined by Ki67-positivity, might not immediately lead to vascular leak, but vessel disruption triggered by endothelial proliferation could occur some time afterwards, eventually resulting in vascular leak. However, by this time, the endothelial cells will have dropped out of cell division and switched off expression of the Ki67 epitope; thus, Ki67 and extravascular fibrinogen leak will never co-localize. To definitively answer this question, we employed an alternative method of identifying proliferating cells, the BrdU labelling technique, in which BrdU was injected intraperitoneally (i.p.) daily for days 0–3 CMH and tissue subsequently stained with anti-BrdU antibodies. The advantage of this technique over Ki67 staining is that it provides more of a long-term record of cell proliferation, in that once a proliferating cell is labelled with BrdU, it remains labelled even after it has stopped dividing [17]. Consistent with our findings using Ki67 staining, CD31/BrdU/fibrinogen triple-IF revealed that BrdU^+^ endothelial cells and fibrinogen^+^ extravascular leaks never co-localized. As shown in Fig. 7A, CD31^+^/BrdU^+^ proliferating endothelial cells (arrows) were never associated with extravascular fibrinogen leaks. In a relatively small number of extravascular leaks, we identified some BrdU^+^ cells (asterisk in lower panel of Fig. 7A), but close inspection revealed that these BrdU^+^ nuclei never co-localized to CD31^+^ endothelial cells (see Fig. 7A high power image), suggesting that another type of central nervous system (CNS) cell is proliferating. Based on our previous findings that extravascular fibrinogen leaks are associated with accumulation of activated microglial cells [10, 18], we wondered if the proliferating cells might be microglia. We tested this by performing Mac-1/BrdU/fibrinogen triple-IF, and as shown in Fig. 7B, the BrdU^+^ nuclei were localized to Mac-1^+^ microglia. Additional staining with Mac-1/Ki67/fibrinogen triple IF confirmed that the proliferating cells associated with extravascular fibrinogen leak were always Mac-1^+^ microglia (Fig. 7C). As pericytes have been shown to play an important regulatory role in the maintenance of the BBB [4], we also examined whether the distribution of pericytes changes around leaky blood vessels by studying the expression of the pericyte marker platelet-derived growth factor receptor-beta (PDGFRβ) in conjunction with CD31 and fibrinogen. This revealed that unlike the microglial aggregation response, there was no obvious accumulation of pericytes around leaky blood vessels; nor was there any major loss of pericytes from leaky vessels (Fig. 7D).

### Tight junction protein expression is maintained in angiogenic blood vessels but lost in a significant proportion of vessels showing extravascular leak

**Fig. 8:**
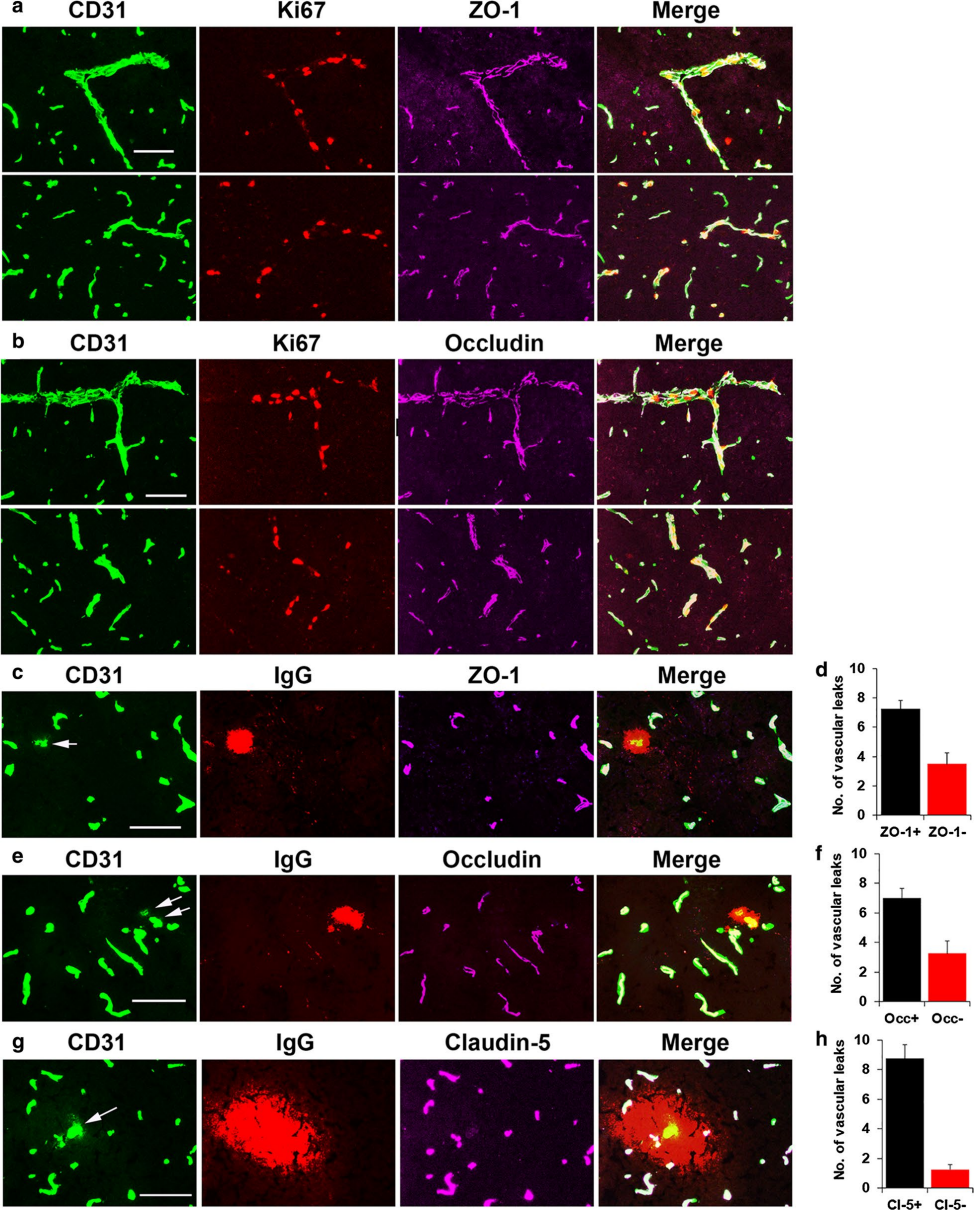
Selective loss of tight junction proteins in leaky vessels but not angiogenic vessels. **A–C, E, G** Frozen brain sections taken from mice exposed to 4 days hypoxia (8% O_2_) were triple-labelled for CD31 (AlexaFluor-488), Ki67 (Cy3) and ZO-1 (Cy5) (**A**), CD31 (AlexaFluor-488), Ki67 (Cy3) and occludin (Cy5) (**B**), CD31 (AlexaFluor-488), IgG (Cy3) and ZO-1 (Cy5) (**C**), CD31 (AlexaFluor-488), IgG (Cy3) and occludin (Cy5) (**E**) or CD31 (AlexaFluor-488), IgG (Cy3) and claudin-5 (Cy5) (**G**). Scale bars = 50 μm. **D**, **F**, **H** Quantification of the number of ZO-1, occludin or claudin-5 positive and negative vessels after 4 days CMH. All results are expressed as the mean ± SEM (n = 4 mice/group). Note that angiogenic vessels maintained their expression of the tight junction proteins ZO-1 and occludin (**A** and **B**), but many leaky vessels lost tight junction protein expression (arrows in **C**, **E** and **G**)

As we found that angiogenic blood vessels containing proliferating endothelial cells do not show extravascular leak, while a small number of non-angiogenic vessels show full-blown extravascular leak, we next examined whether loss of tight junction proteins, a key component of the BBB, differs in these two situations. Triple-IF with CD31/Ki67/ZO-1 or CD31/Ki67/occludin showed that angiogenic vessels containing proliferating endothelial cells maintained their vascular expression of the tight junction proteins ZO-1 and occludin. This is shown both in larger vessels (upper panels), as well as capillaries (lower panels) of Fig. 8A and B. Similar results were found for claudin-5 (not shown). In contrast, CD31/IgG/ZO-1, CD31/IgG/occludin or CD31/IgG/claudin-5 triple-IF, employing IgG as a marker of extravascular leak, revealed that in some vessels showing extravascular leak, marked loss of tight junction proteins occurred (see arrows in Fig. 8C, E, G). Quantification revealed that approximately one-third of all vessels showing extravascular IgG leak had obvious loss of ZO-1 or occludin, though interestingly, loss of claudin-5 was only evident in a smaller fraction of leaky blood vessels (Fig. 8D, F, H).

## Discussion

While it has been known for many years that chronic mild hypoxia (CMH, 8% O_2_) promotes a strong vascular remodelling response in the brain, resulting in greater vessel density [8, 9], only recently has it been shown it also provokes transient vascular disruption in a small number of blood vessels [10, 18]. Based on our recent finding that on a region-to-region basis, the extent of cerebrovascular leak correlates closely with the degree of angiogenic remodelling, we wanted to answer the fundamental question: is the vascular leak an unwanted side-effect of angiogenic remodelling or is it a separate pathological response, in which hypoxic insult triggers endothelial dysfunction? In the current study, we addressed this question by performing a detailed spatial and temporal immunofluorescent analysis of CMH-induced cerebrovascular remodelling. Our main findings were as follows: (1) most endothelial proliferation and extravascular leak occurred in capillaries and to a lesser degree in venules, (2) surprisingly, endothelial proliferation and extravascular leak never colocalized, (3) interestingly, endothelial proliferation strongly colocalized with an intravascular fibrinogen staining pattern, (4) DiI perfusion studies showed that angiogenic vessels were well perfused, suggesting that fibrinogen retention in angiogenic vessels is not due to transient closure of the vessel, but more likely, because fibrinogen is retained within the vessel wall, (5) using BrdU labelling as a means of more permanently labelling proliferating cells, we confirmed lack of any connection between mitogenic endothelial cells and extravascular fibrinogen leak, (6) while in contrast, when proliferating cells were found in extravascular leaks, they were always microglia. Taken together, these data suggest that extravascular leak is not an unwanted side-effect of angiogenic remodelling, but rather a dysfunctional hypoxic response that occurs in a small distinct subset of non-angiogenic blood vessels.

### Most endothelial proliferation and vascular leak occur in capillaries

In this study we made use of the previous finding that laminin-111, a component of the leptomeningeal basement membrane in the brain, labels only venous and arterial vessels, but not capillaries [13]. As α-SMA identifies arterial vessels, it becomes relatively easy to distinguish the three different types of blood vessel using these markers. Its perhaps not so surprising that most of the extravascular leak occurred in capillaries, because in contrast to arterioles and venules, which contain high and low amounts of smooth muscle cells respectively, capillaries have none and thus have the weakest walls. Some vascular leaks were spatially associated with venules, though because the area of extravascular leak often extended over several different blood vessels, it is hard to know whether this truly represents venular leak per se, or whether a leak that originated in a capillary happened to extend as far as a neighboring venule. However, venular leak would be consistent with the well-described loss of BBB integrity in inflammatory demyelinating disease, in which leukocytes preferentially infiltrate the CNS through post-capillary venules [13]. Notably, vascular leak was never detected in arterioles. In the same vein, endothelial proliferation occurred predominantly in capillaries (approximately 67%) and to a lesser degree in venules (approximately 33%), but only exceedingly rarely in arterioles. Its currently unclear why CMH stimulates such a strong endothelial proliferation response in capillaries and venules but not arterioles. This was unexpected in light of our previous finding that CMH induces a marked arteriogenic response as indicated by an increased number of α-SMA+ vessels [14]. One possibility is that while endothelial proliferation would not be disastrous in the low-pressure capillaries and venules, such remodelling in the relatively higher-pressure arterioles could trigger devastating vascular leak. By extension, its plausible that arteriogenic remodelling may be underpinned more by hypertrophic responses of a relatively fixed number of endothelial cells rather than the hyperplastic/proliferation response seen in capillaries and venules.

A series of studies from the LaManna lab have described some of the signalling pathways thought to be important in mediating the CMH-induced angiogenic response. Based on these studies, our current understanding is that hypoxia stimulates upregulated expression of hypoxia-inducible factor-1α (HIF-1α) [19], which then triggers vascular endothelial growth factor (VEGF) and angiopoietin 2 production in astrocytes and pericytes [20–22]. These factors then stimulate receptors on endothelial cells, which promotes endothelial uncoupling from neighboring cells and switches endothelial cells into a proliferative and migratory phenotype, facilitating angiogenic expansion of the vascular network [23]. In addition, studies from our lab have demonstrated an important role for the ECM protein fibronectin, which is strongly upregulated in the vascular basement membrane in response to hypoxia. This is accompanied by increased expression of the fibronectin receptor α5β1 integrin on angiogenic endothelial cells [24]. Genetic knockout studies have revealed that absence of the α5β1 integrin specifically on endothelial cells results in an attenuated angiogenic response [25]. Further studies suggest that fibronectin stimulation of endothelial cells may be mediated via activation of the MAP kinase signalling pathway [26].

### Uncoupling of angiogenesis and vascular leak

At the outset of these studies, we predicted that angiogenic blood vessels would show transient leak as they underwent remodelling. However, the major take home message from this work is that endothelial proliferation and vascular leak occur in totally different vessels, suggesting that extravascular leak is not an unwanted side-effect of angiogenic remodelling, but rather a dysfunctional hypoxic response that occurs in a small distinct subset of non-angiogenic blood vessels. It’s important to realize that at any given time, the number of leaky cerebral blood vessels is relatively small (<1% vessels), and even more importantly, our studies show that at this level of hypoxia (8% O_2_), the leaks tend to resolve over time, as shown by reduced numbers of vascular leaks at the later (day 14) time point. This clearly demonstrates that cerebral blood vessels are efficient at repairing vascular disruption under these conditions.

Our studies raise the obvious question: why does hypoxia trigger BBB disruption; what’s the mechanism? If its not a side-effect of angiogenic remodelling, then why do they leak? One possibility is that vascular disruption is the result of failure to mount an effective angiogenic response. As hypoxia drives the angiogenic response in an attempt to meet the metabolic demands of neural tissue, it is conceivable that a failure to meet this demand will lead to cellular hypoxia and endothelial dysfunction, which would then result in loss of vascular integrity. This sequence of events occurs in the extreme hypoxic situation of ischemic stroke, in which lack of oxygen triggers endothelial dysfunction and loss of vascular integrity, often resulting in irreversible vascular damage, especially at the ischemic core [27]. In this light, its worth considering two observations. First, while the ischemic core is initially limited to a small region surrounded by an ischemic border or penumbra, over time, this core expands out to encroach into the penumbra, resulting in a much larger ischemic core. Second, blood vessels within the ischemic penumbra launch an angiogenic response, presumably to meet the increasing demand for blood in the ischemic region. Based on this, it seems likely that shortly after vessel occlusion, blood vessels in the ischemic core will be too devoid of oxygen and nutrients to launch an angiogenic response and will start to break down and leak and undergo degeneration. By contrast, vessels in the penumbra, which will still be receiving a sufficient supply of blood to support remodelling, will at this time, activate the angiogenic response to increase tissue vascularity in this region. However, in the ensuing days, as the level of perfusion to more distal regions of the ischemic penumbra gradually becomes more restricted, these vessels will also start to break down and degenerate.

The concept that failure to mount an adequate angiogenic response triggers vascular leak is further supported by our previous demonstration that transgenic mice lacking endothelial expression of the angiogenic α5 integrin receptor (α5-EC-KO mice) showed attenuated endothelial proliferation and much greater vascular breakdown in an experimental autoimmune encephalomyelitis (EAE) model of multiple sclerosis [28]. In future experiments we plan to test this idea more fully, both by studying CMH-induced vascular leak in α5-EC-KO mice and by blocking angiogenesis via inhibition of the VEGF signalling pathway.

While our findings offer important insight into the events underlying hypoxia-induced cerebrovascular remodelling, the design of the current study have several limitations. First, we studied these events at only one level of hypoxia (8% O_2_). As this level of hypoxia is generally regarded as sub-clinical, it would be interesting to study more severe levels of hypoxia that approximate closer the extreme level of hypoxia attained during ischemic stroke to examine if the relationship between angiogenesis and vascular leak is any different under conditions of more extreme hypoxia. Second, the markers of BBB breakdown used in this study were relatively large (IgG and fibrinogen having Mr of approximately 150 and 340 kD respectively). In future studies it will be insightful to study these processes using smaller molecular weight markers, so as to examine whether smaller leaks occur in a larger proportion of blood vessels, and also to see if angiogenic vessels, while not showing large leaks, show any relative loss of vascular integrity. Third, it will be important to determine if the processes of hypoxia-induced cerebrovascular leak and angiogenesis are affected by age. Lastly, in this study, while we described which type of blood vessel hypoxia-induced vascular leak occurs in, and its time-course, other than a brief analysis of tight junction protein expression and its disappearance on leaky vessels, we did not specifically address the relative roles of paracellular vs. transcellular permeability. We are currently addressing some of these important questions in ongoing studies.

### What’s the biological significance of intravascular fibrinogen deposition in angiogenic vessels?

A significant finding of this study was the presence of fibrinogen deposition within the walls of angiogenic blood vessels. We first hypothesized that this might be a result of a coordinated temporary vessel closure to protect the remodelling vessel as the endothelial cells temporarily break their cell-cell contacts to proliferate and reposition to extend the vascular network, but our DiI perfusion studies discounted this possibility by demonstrating normal perfusion of the remodelling vessels. While our studies clearly demonstrate that vessels showing the intravascular fibrinogen staining pattern do not progress to those showing full-blown extravascular leak, it is still possible that intravascular fibrinogen deposition represents a partial vascular disruption, coincident with neighbouring endothelial cells separating from their neighbours. According to this idea, fibrinogen would leak through the first stage of the BBB barrier, i.e., through the partially separated endothelial cells, but not get beyond the second line of defence, namely the vascular basal lamina. An alternative explanation is that fibrinogen deposition in the wall of angiogenic blood vessels is part of a coordinated physiological response in which fibrinogen promotes the angiogenic process. In recent studies we have described a critical role for fibrinogen in stimulating microglial recruitment, activation and proliferation around leaky cerebral blood vessels [10, 18], so it conceivable that fibrinogen deposits in the vessel wall play a role in activating and stimulating endothelial proliferation as part of the angiogenic process. In future studies, we plan to investigate this possibility by studying these hypoxia-induced remodelling events in fibrinogen KO mice [29].

## Conclusions

CMH stimulates robust vascular remodelling in the brain, as well as transient vascular disruption. In this study we have addressed the fundamental question: is the vascular leak an unwanted side-effect of angiogenic remodelling or is it a pathological response, unrelated to endothelial proliferation, in which declining oxygen levels trigger endothelial dysfunction? Our findings demonstrate that endothelial proliferation and extravascular fibrinogen leaks never co-localize, suggesting that extravascular leak is not an unwanted side-effect of angiogenic endothelial proliferation, but rather a dysfunctional vascular response to hypoxia that occurs in a distinct group of non-angiogenic blood vessels.

## Data Availability

The datasets used and/or analysed during the current study are available from the corresponding author on reasonable request.
